# Evaluation of Nematicidal Activity of *Streptomyces yatensis* KRA-28 against *Meloidogyne incognita*

**DOI:** 10.4014/jmb.1908.08038

**Published:** 2020-03-27

**Authors:** Eun-Jae Park, Hyun-Jae Jang, Chan Sun Park, Seung-Jae Lee, Soyoung Lee, Kang-Hoon Kim, Bong-Sik Yun, Seung Woong Lee, Mun-Chual Rho

**Affiliations:** 1 Immunoregulatory Materials Research Center, Korea Research Institute of Bioscience and Biotechnology (KRIBB), Jeongeup 5622, Republic of Korea; 2 Division of Biotechnology and Advanced Institute of Environment and Bioscience, College of Environmental and Bioresource Sciences, Jeonbuk National University, Iksan 54596, Republic of Korea

**Keywords:** *Meloidogyne incognita*, *Streptomyces yatensis* KRA-28, *Streptomyces misionensis* KRA-24, biocontrol, nematicidal activity

## Abstract

The root-knot nematode (*Meloidogyne incognita*) is an important pathogen in crop cultivation, however, few methods are available to control this parasitic roundworm. In this study, the nematicidal effects of approximately 30 *Streptomyces* strains isolated from soil samples of Mt. Naejang (Korea) were tested against *Meloidogyne incognita*, and the culture broth of the strains KRA- 24 and KRA-28 exhibited approximately 75% and 85% insecticidal activity, respectively, in in vitro assays. In in vivo pot experiments, these strains reduced the number of nematodes in the soil and the number of egg masses in the roots of red peppers. The two strains also survived in the presence of insecticidal agents (0.1 to 3.0%) such as fosthiazate, ethoprophos and terbufos when they were used in parallel. The mixture of KRA-24 or KRA-28 culture broth and fosthiazate exhibited nematicidal effects that were similar to those observed when KRA-24 or KRA-28 were used alone. Our results clearly suggest that the *Streptomyces* strains KRA-24 and KRA-28 should be promoted as a biocontrol agent against *Meloidogyne incognita*.

## Introduction

The loss of agricultural products to root-knot nematodes has been increasing annually worldwide. Root-knot nematodes belong to the genus *Meloidogyne* and are one of the most important causes of damage to major cultivated crops (pepper, potato, sweet potato, tomato, okra, cabbage, lettuce, etc.) [1, 2]. Larvae of root-knot nematodes initially infect the roots of various crops, including watermelons, tomatoes, and carrots, and subsequently produce egg masses on the roots, resulting in root dysfunction that reduces the efficiency of plant water and nutrient utilization [1]. Therefore, infection by root-knot nematodes can cause significant decreases in plant yields and survival as a result of stunted root growth, root necrosis, and from increased susceptibility to various plant diseases [1, 3]. Root-knot nematodes are distributed worldwide, and *M. incognita*, *M. arenaria*, *M. hapla*, *M. cruciani*, *M. javanica* and *M. hispanica* have been reported in Korea [4], where they severely damage watermelon, melon, tomato and oriental melon crops [5].

In general, a variety of methods are used to limit *Meloidogyne* damage, including the development of resistant crops, the control of cultivation environments (improvement of soil and sterilization by sunlight), the treatment of plant diseases using chemical nematicides, and the biological control of root-knot nematodes using microorganisms or plant extracts [6-11]. Among these methods, the control of root-knot nematodes by chemical nematicides is most heavily relied upon because the use of resistant crop varieties and controlling cultivation environments are methods with lower efficacy. However, chemical agents, including fosthiazate, ethoprophos and terbufos, have severely deleterious effects on the environment, human health and beneficial microorganisms in soil [12]. Thus, interest in the development of new nematicidal agents that do not impact crops, farmers, consumers, or the environment is growing. Accordingly, the use of the culture broth or secondary metabolites of microorganisms as biological control agents is an effective alternative to using chemical nematicides for the management of plant diseases [13]. Furthermore, the broad spectrum of disease suppression activities of microorganisms and their secretions and the possibility of combining their use with other control methods may promote their increased usage. In particular, *Streptomyces* is the main genus of actinomycetes that has been found to be active against nematodes by antagonism or parasitism in many studies [13-15]. Kim *et al*. (2012) reported that *Streptomyces cacaoi* GY525, isolated from liquid compost containing crab shell powder, could parasitize eggs of *M. incognita*, inhibit the egg hatch, and kill second-stage juveniles (J2s) in vitro. Also, Ruanpanun *et al*. (2011) tested the nematicidal activity of *Streptomyces* sp. CMU-MH021 on second-stage juveniles of *M. incognita*, and reported that the strains inhibited the egg hatch and induced *M. incognita* larval mortality in vitro. Therefore, we selected *Streptomyces* strains and conducted studies to derive anti-nematode active strains for *Meloidogyne incognita*.

In the search for a biological agent to control root-knot nematodes, we isolated various *Streptomyces* strains from soil samples and tested their potential insecticidal activity against *Meloidogyne incognita*. *Streptomyces yatensis* strain KRA-28 exhibited the most robust insecticidal activity of the tested strains, and its nematicidal potential was evaluated through in vivo pot experiments using red pepper plants.

## Materials and Methods

### Isolation of *Streptomyces* spp. from Soil

*Streptomyces* spp. were isolated from soil samples obtained from Mt. Naejang in Korea. For each collected soil sample, 1 g of soil was pretreated in a 100°C oven for 1 h and then diluted in 100 ml of a saline solution (0.85%NaCl). Five different dilutions (1:10, 1:100, 1:1,000, 1:10,000, and 1: 100,000) were prepared using saline solution, from which 0.1 ml aliquots were plated on Bennett’s agar medium [16] as a selective medium. The plates were incubated at 28°C for 96 h, after which colonies were selected and transferred to fresh Bennett’s agar medium. Isolated *Streptomyces* spp. were stored at -80°C in 20% (v/v) glycerol until use. For in vitro and in vivo experiments, the isolates were cultured on modified GSS medium (10 g soluble starch, 20 g dextrose, 4 g yeast extract, 1 g beef extract, 2 g NaCl, 0.25 g K_2_HPO_4_, 2 g CaCO_3_, and 25 g defatted soybean powder, w/v) at 28°C for 72 h [15].

### Identification of Isolated Strains

A 16S ribosomal RNA sequencing analysis was performed according to the manufacturer’s protocol (Genotech Co., Korea). DNA extracted from actinomycetes using a Genomic Cell/Tissue Spin Mini Kit according to the manufacturer’s instructions (Nucleogen). The 16S rRNA gene was amplified by PCR using two universal primers, 9F (5'-GAGTTTGATCCTGGCTCAG-3') and 1512R (5'-ACGGTTACCTTGTTACGACTT-3'), which were sequenced using an ABI Prism 3730xi Sequencer. The 16S ribosomal RNA gene sequences were compared with those in the GenBank database using the program BLAST.

### Nematode Culturing

*Meloidogyne incognita* was obtained from egg masses on the roots of red pepper plants (*Capsicum annuum* cv. Bugang) using the method of Moon *et al*. (2010) [17]. First, red pepper plants grown for 4 weeks were infected with 1,000 *Meloidogyne incognita* juveniles, which were obtained from Prof. Young Ho Kim (Seoul National University, Korea). After 60 days, the plants were uprooted from pots, washed with flowing water, and the egg masses on the roots were harvested. The egg masses were incubated in sterile water for three days at r.t. in a Baermann funnel, and the hatched second-stage juveniles (J2s) were subsequently harvested and used for nematicidal activity and in vivo pot experiments.

### Nematicidal Activity

The nematicidal activity of the culture broth from each strain was determined using the J2s [18]. A fresh suspension of J2s (100 J2s/100 μl) was added to the wells of a 96-well plate and treated with culture broth at concentrations of 10, 5, 3, and 1%. Sterilized GSS broth or sterile distilled water was used as negative control. Five replicates were performed for each culture broth. The plates were incubated at 25°C for 24 h, and the number of living and dead juveniles were counted using a microscope at 6, 12, and 24 h. The experiment was performed with five replicates. Mortality was calculated according to the following formula: juvenile mortality = 100 × dead juveniles/total juveniles.

### Survival Test

We also conducted experiments to confirm whether the isolated strains could survive on Bennett’s medium containing anti-nematode agents, such as fosthiazate (Seonchungtan, Korea), ethoprophos (Mocap, Korea) and terbufos (Kaunta, Farm Hannong, Korea). After the concentration of anti-nematode agents in Bennett’s medium was adjusted to 0.1-5%, the isolated strains were inoculated at a concentration of 1.5 × 10^2^ cells/ml. The flasks were incubated at 28°C for 72 h, and the growth of the strains was measured. Next, 0.1 ml of culture medium was spread on Bennett’s agar plates; the inoculated plates were then incubated at 28°C for 96 h, and the colonies grown were counted. The experiment was performed with five replicates.

### In Vivo Effect of KRA-24 and KRA-28 in Pot Experiments

In vivo pot experiments were conducted to investigate the antagonistic activity of *Streptomyces misionensis* KRA-24 and *Streptomyces yatensis* KRA-28 on *Meloidogyne incognita*. The experiments were performed in a controllable greenhouse that was maintained at 25 ± 2°C and 60 ± 2% humidity with a 12 h light and dark cycle. Red pepper seeds were planted in pots containing nursery soil (Bunong horticulture nursery soil, Korea). Two-week-old red pepper seedlings were transplanted into plastic pots with a 6-cm diameter containing 500 g of sterilized sand and potting mixture. After the plants were cultivated for four weeks, they were inoculated with approximately 1000 *M. incognita* J2s [19]. Four weeks after inoculation, the J2s-infected plants were divided into 7 groups, and each group was given a different treatment. Group 1: each plant was inoculated with GSS medium without microbial cultures. Groups 2 and 3: each plant was treated with 100 ml of a 10-fold dilution of culture broths of *S. misionensis* KRA-24 or *S. yatensis* KRA-28, which was repeated every 2 weeks. Group 4: each plant was treated with 100 ml of an aqueous solution containing 9.5 mg of an anti-nematode agent, and these plants served as a positive control. Groups 5 and 6: each plant was inoculated with 100 ml of anti-nematode solution and 100 ml of the 10-fold diluted culture broths of *S. misionensis* KRA-24 and *S. yatensis* KRA-28. Group 7: each uninfected plant was treated with 100 ml of water, and these plants served as a negative control. Each pot experiment was replicated five times. Plants were watered using 100 ml/pot every 4 days, and after 30 and 60 days, the length and dry weight of roots and shoots, the number of egg masses on roots, and the number of nematodes in the soil were measured.

The number of egg masses on roots was determined using the phloxine B staining method [20]. In addition, the number of bacteria, including actinomycetes, in the soil was determined using the modified funnel method [21]. Briefly, 1 g of soil was diluted 10-fold with a 0.85% NaCl solution, after which the mixture was plated on LB agar plates. The plates were incubated for 2 days at 30°C, and the number of colonies was subsequently determined. In the case of actinomycetes, 1 g of soil was heated at 100°C for 1 h and then diluted 10-fold in 0.85% NaCl. The diluted mixture was then plated on Bennett’s medium agar plates and incubated at 28°C for 4 days, and the number of colonies were then counted.

### Data Analysis

Statistical analyses were performed using one-way analysis of variance, and the means of the treatments were determined by Duncan’s multiple-range test (*p* < 0.05) using SPSS (version 16.0 for Windows; SPSS, USA).

## Results

### Selection of Anti-Nematode Strains

**Fig. 1:**
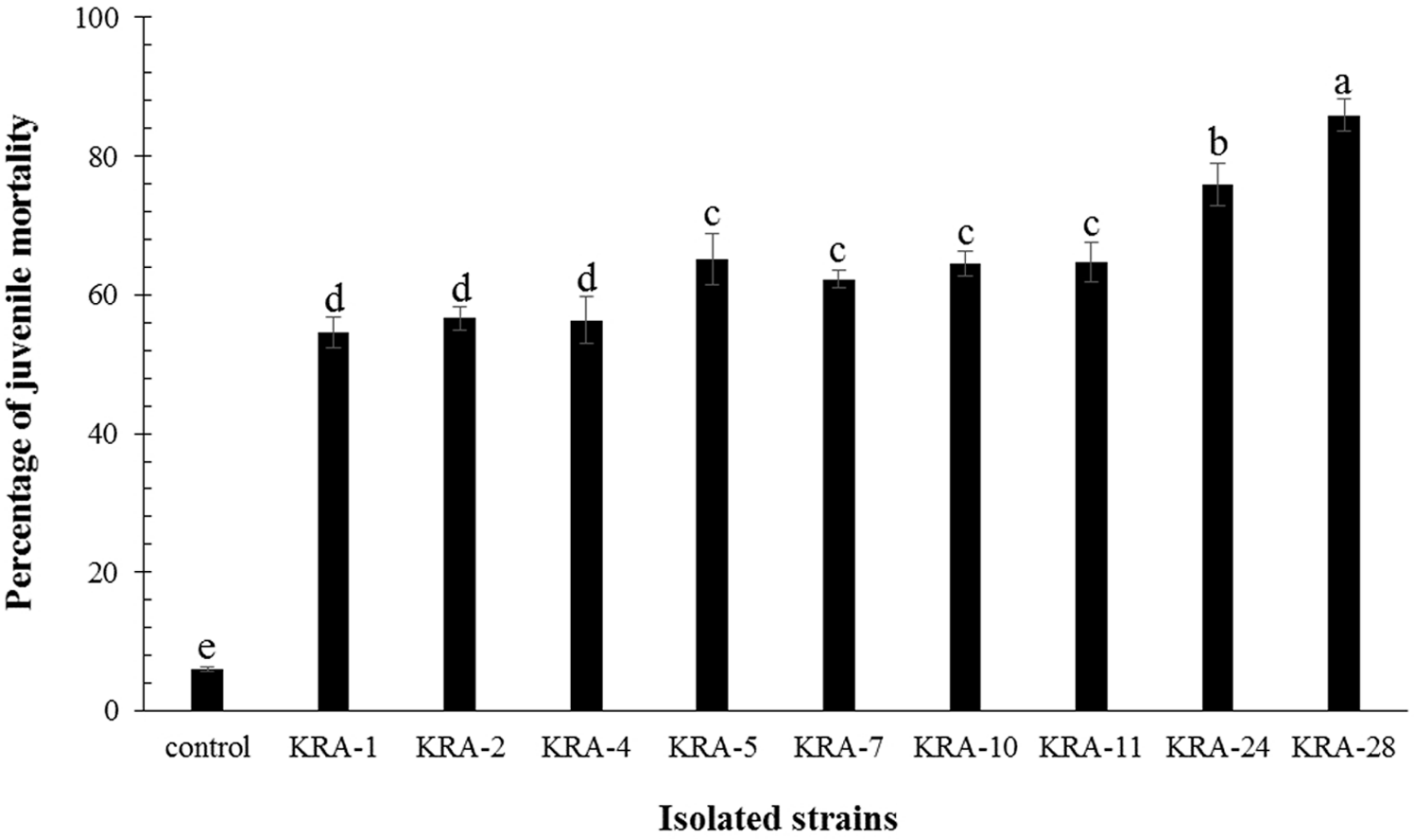
In vitro nematicidal activity of nine isolated strains. The experiment was performed with five replicates. Water was used as the control. Values with the same letter on the bar do not significantly differ (*p* < 0.05) based on Duncan’s multiple range test.

Thirty *Streptomyces* strains isolated from soil samples of Mt. Naejang in Korea were tested for anti-nematode activity against *Meloidogyne incognita*. *M. incognita* J2s were treated with the culture broths of the 30 isolated strains at a concentration of 10%, and the viability of the J2s was determined after a 48 h incubation. Among the isolated strains, the culture broths of nine strains caused over 50% mortality of J2s. As shown in Fig. 1, an 85.9 ± 2.3% mortality of J2s was observed in the group treated with culture broth of the KRA-28 strain, and for the group treated with the KRA-24 strain, a 75.9 ± 3.1% mortality of J2s was observed. For the KRA-5, KRA-7, KRA-10 and KRA-11 culture broths, the observed mortality of J2s was 65.2 ± 3.6%, 62.3 ± 1.3%, 64.5 ± 1.9%, and 64.8 ± 2.8%, and for the groups treated with KRA-1, KRA-2 and KRA-4 culture broths the number of J2s was reduced by 54.5 ± 2.3%, 56.7 ± 1.7%, and 56.4 ± 3.4% (Fig. 1).

**Fig. 2:**
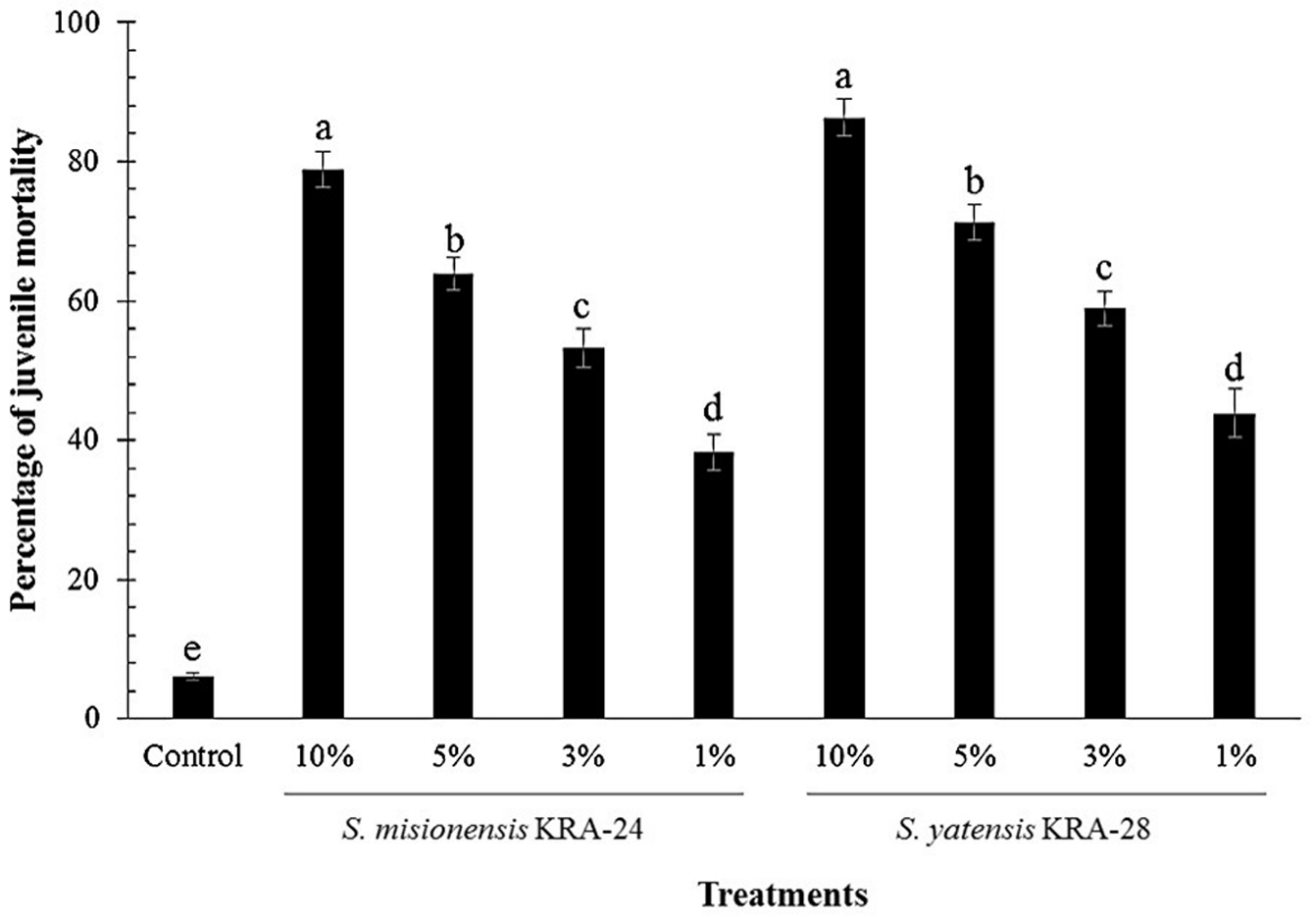
In vitro nematicidal effect of *Streptomyces misionensis* KRA-24 and *Streptomyces yatensis* KRA-28. Plants were treated with culture supernatants at concentrations of 10, 5, 3, and 1%. The experiment was performed with five replicates. Water was used as the control. Values with the same letter on the bar do not significantly differ (*p* < 0.05) based on Duncan’s multiple range test.

To further determine the larvicidal potential of biocontrol agents, we tested different concentrations of culture broths from strains KRA-24 and KRA-28 against *M. incognita* J2s. The mortality rate of J2s treated with KRA-28 culture broth scaled in a dose-dependent manner, increasing from 43.9 ± 3.5 to 86.3 ± 2.7% as the concentration of culture broth increased from 1 to 10% (Fig. 2). The J2s were also significantly affected by KRA-24 culture broth, which induced mortalities of 38.3 ± 2.6, 53.2 ± 2.8, 63.9 ± 2.4, and 78.9 ± 2.5% at concentrations of 1, 3, 5, and 10%, respectively, after a period of 48 h. Thus, the KRA-28 strain culture broth showed an approximately 10% greater anti-nematode activity in the larvicidal assay than that of the KRA-24 strain (Fig. 2). KRA-28 strain was identified as *Streptomyces yatensis* KRA-28, with closest 16S rDNA sequence identity (99%) to that of the type strain *Streptomyces yatensis* strain DSM 41771 by analyzing their 16S ribosomal RNA gene sequences. KRA-24 strain was identified as *Streptomyces misionensis* KRA-24.

### Viability of the KRA-24 and KRA-28 Strains in Medium Containing Anti-Nematode Agents

To assess whether *S. misionensis* KRA-24 and *S. yatensis* KRA-28 could be used as biocontrol agents in the field, we measured the viabilities of these strains in medium containing insecticides, such as fosthiazate (Seonchungtan), ethoprophos (Mocap) and terbufos (Kaunta), which are marketed as anti-nematode agents in Korea. Each strain was inoculated with 1.5 × 10^2^ in Bennett’s medium containing 0.1-5.0% insecticide and incubated at 28°C for 72 h. As shown in Table 1, the growth of two strains was not affected at insecticide concentrations of 0.1-0.15%, which are the recommended concentrations for use of these agents. In addition, the two strains grew in the range of 10^2^-10^4^ in the presence of 0.5-3.0% fosthiazate, ethoprophos and terbufos, but not at 5%.

### Effects of KRA-24 and KRA-28 on Plant Growth and the Number of Nematodes and Egg Masses

Pot experiments were carried out to further assess the in vivo biocontrol efficacy of *S. misionensis* KRA-24 and *S. yatensis* KRA-28 and to select a strain for use in the field. The red pepper seedlings were planted in pots and then infected with J2s for four weeks. After the plants were confirmed to be infected with *M. incognita*, culture broths of the strains KRA-24 and KRA-28 were applied to the plant at 100 ml/pot every two weeks. After 60 days, the length and dry weight of shoots were 28.4 ± 1.1 cm and 0.65 ± 0.04 g, respectively, whereas in the control (only infected J2s) and negative control groups (treated only with H_2_O), these values were 32.8 ± 2.2 cm and 0.92 ± 0.03 g. The group treated with the culture broth of strain KRA-28 exhibited a greatly enhanced shoot length (4.4 cm) and dry weight (0.18 g). In addition, treatment of plants with the culture broth of strain KRA-24 increased the shoot length by nearly 1.6 cm, resulting in similar dry weight values to those observed in the infected control group. However, the root length and dry weight of red pepper plants in the group treated with KRA-24 and KRA-28 did not increase significantly. The results indicated that all treatments increased the length and dry weight of shoots, and these data are presented in Table 2.

As shown in Table 3, all treatments significantly reduced the number of juveniles and egg masses at 30 and 60 days after inoculation compared to the values observed for the infected control group (infected only with J2s). After 60 days, the number of nematodes and egg masses in the infected control group increased, totaling 1836 ± 84.4 nematodes and 142.8 ± 5.3 egg masses. The greatest reduction in the number of egg masses (total of 42.2 ± 4.8 egg masses) and the population of nematodes (total of 542 ± 48.2 nematodes) was observed when the culture broth of strain KRA-28 was applied (Table 3). Additionally, compared with that of strain KRA-28, the culture broth of strain KRA-24 moderately reduced the number of egg masses (total of 73.8 ± 4.6 egg masses) and the nematode population in soil (total of 644 ± 32.9 nematodes) (Table 3).

### Effects of Using a Combination of KRA-24 or KRA-28 and Anti-nematode Agent

Experiments were conducted to determine whether *S. misionensis* KRA-24 and *S. yatensis* KRA-28 could be applied to soil containing pesticides. After red pepper plants were treated with fosthiazate and cultures of KRA-24 or KRA-28, the changes in the roots and shoots of crops and the number of nematodes in soil and egg masses on roots were measured. After 60 days (Table 2), the shoot length of the group treated with fosthiazate had increased by 3.8 cm, however, the dry weights of the shoots and roots of plants in this group were reduced by 0.15 ± 0.02 g and 0.47 ± 0.05 g, respectively. These results showed that the recommended usage of this agent (0.1%) affected the growth of crops. The number of nematodes (total of 492 ± 135.9 nematodes) and egg masses (total of 14.4 ± 3.9 egg masses) of the group treated with fosthiazate was reduced by 73.2 and 89.9% compared with the control group infected with J2s, respectively (Table 3). As shown in Table 3, the combined use of fosthiazate and KRA-24 or KRA-28 reduced the number of nematodes and egg masses by 72.5 ± 2.3-73.3 ± 5.9% and 88.9 ± 3.9-92.9 ± 3.5% compared with the control group infected with J2s, respectively. In particular, this combination considerably improved the dry weight of roots and shoots compared with those of the group treated with fosthiazate alone (Table 2).

### Analysis of Microbial Changes in Soil Treated with KRA-24 and KRA-28

We also measured the change in the number of total bacteria and actinomycetes in the soils treated with *S. misionensis* KRA-24 and *S. yatensis* KRA-28. In the control group infected with J2s, the number of initial bacteria was approximately 10^3^ and increased by 10- and 100-fold after 30 and 60 days, respectively. Compared with the control group infected with J2s, the number of bacteria in the group treated with fosthiazate decreased by approximately 100-fold after 30 and 60 days. Additionally, the total number of bacteria in the group treated with KRA-24 or KRA-28, compared to that in the control group infected with J2s, decreased by approximately 10-fold. Table 4 shows that there was no significant difference in the number of bacteria in the microbe-treated groups and those treated with both fosthiazate and KRA-24 or KRA-28. This result showed that the KRA-24 or KRA-28 strains can survive in soil for a long time without being affected by fosthiazate.

## Discussion

The most commonly used method to control root-knot nematodes is the application of chemicals, such as fosthiazate, terbufos and ethoprophos. However, the use of chemical agents is limited due to their ability to cause human toxicity, soil pollution, water pollution, and the emergence of resistant bacteria [12]. Biological agents that control nematodes have been actively searched for in recent years, and several plant extracts and microorganisms have been successfully applied to plant roots [9, 10, 22]. However, these approaches have been less effective than organic synthetic pesticides, and these biocontrol agents are less widely available as eco-friendly controls. Biological control agents, such as *Myrtus communis*, *Nepeta cataria*, *Tagetes patula*, *Zoysia japonica*, *Rhus sylvestris*, *Rhus chinensis* and *Allium cepa* have been useful in managing root-knot nematodes [10, 23, 24]. Additionally, several microorganisms, such as *Paenibacillus lentimorbus*, *P. polymyxa*, *Bacillus thuringiensis*, *B. megaterium*, *Trichoderma harzianum* and *Streptomyces* spp. have been used to study nematode control [19,25-28]. *Streptomyces* spp., including *S. cacaoi*, *S. avermitilis*, and *S. hydrogenans* have been particularly used to control plant parasitic nematodes in many studies [12, 14, 29] because they produce large amounts of antibiotic compounds, including streptomycin, neomycin, fosfomycin, tetracycline, kanamycin, and vancomycin. Therefore, there is a need to develop biological agents using microorganisms that have no serious chemical risks and have a good impact on soil and crops.

In our present study, we identified a novel *Streptomyces* strain with excellent anti-nematode activity that is applicable to environmentally friendly agriculture. As a result of screening for anti-nematode activity, two strains, KRA-24 and KRA-28, were observed to cause 85.9 and 75.9% mortality rates of J2s after a 48 h incubation, respectively. Additionally, the culture broth of KRA-28 strain showed dose-dependent anti-nematode activity and an approximately 10% higher anti-nematode activity than that of KRA-24 (Fig. 2). These strains were identified as *Streptomyces misionensis* KRA-24 and *Streptomyces yatensis* KRA-28 by analyzing their 16S ribosomal RNA gene sequences. Two strains were selected for study because they may behave differently when applied to fields, and we conducted pot experiments to confirm that both microorganisms were applicable as biological control agents. When soil is infected by root-knot nematodes, most farmers will use chemical agents to temporarily control the parasites. Accordingly, we conducted experiments to confirm whether these strains were resistant to chemical agents such as fosthiazate. As shown in Table 1, the strains were not greatly influenced by the recommended concentration (0.1%) for use of each chemical agent, and their growth was also not greatly affected at a concentration of 0.5%. When the group treated with microorganisms alone and the group treated with both microorganisms and chemical agent are compared (Tables 1-4), the results show that the growth of microorganisms treated in the early stages was unaffected by the chemical agent. Chemical agents containing fosthiazate are generally used at 0.1% concentrations (for nematode control) in greenhouses, but based on the results in this study using red pepper plants (Table 2), the recommended concentration of this agent (0.1%) negatively affected the growth rate of crops. However, treatment with the *S. misionensis* KRA-24 or *S. yatensis* KRA-28 strain with fosthiazate improved the plant growth over treatment with fosthiazate alone. That is, these strains can be effectively used because they can reduce the growth inhibition effect of chemical agents on the crops without affecting the anti-nematode activity of the chemical agent (Tables 2 and 3). These results demonstrate that these strains can be used with pesticides or can survive in soil where pesticides are present. Recently, Huang *et al*.(2016) reported that using a combination of *Syncephalastrum racemosum* and *Paecilomyces lilacinus* reduced the number of nematodes and the amount of root galling by *Meloidogyne incognita* and increased the root length, shoot length and plant weight of cucumber plants compared with an untreated control group. Additionally, Kaur *et al*. (2016) observed that culture filtrate of *Streptomyces hydrogenans* DH16 and metabolites produced by this strain inhibited hatching of *M. incognita* J2s and killed hatched juveniles. Furthermore, the culture supernatant and cells of this strain effectively controlled root galls and egg masses in nematode-infested tomato plants. However, these two studies were conducted to confirm the inhibition of the nematode activity by pretreatment or simultaneous treatment with microorganisms. Instead, we first confirmed that egg masses formed after infecting the crops with root-knot nematodes, after which we conducted an experiment to verify whether these strains inhibited secondary infection by root-knot nematodes that had hatched from the egg masses. *S. yatensis* KRA-28 was more effective than KRA-24 in reducing the numbers of nematodes and egg masses, and the KRA-28 strain, compared with the KRA-24 strain, greatly enhanced shoot length and dry weight. As a result, we will conduct research on whether *S. yatensis* KRA-28 can be used as a biological control agent in the future.

In conclusion, 30 *Streptomyces* spp. strains isolated from Mt. Naejang soil were tested in larvicidal assay using *M. incognita*, and the culture broth of the KRA-28 strain, compared with that of other strains, exhibited excellent insecticidal activity. This strain reduced the number of nematodes in the soil and the number of egg masses on red pepper roots in pot experiments. Additionally, KRA-28 strain survived in the presence of insecticide agents (0.1 to 3.0%) such as fosthiazate, ethoprophos and terbufos. In future investigations, we will conduct safety and field experiments to test *S. yatensis* KRA-28 as a new biocontrol agent against *Meloidogyne incognita*.

## Figures and Tables

**Table 1:** Insecticide resistance of *S. misionensis* KRA-24 and *S. yatensis* KRA-28.^a^

Treatment ^b^	Concentration (%) ^c^	KRA-24	KRA-28
Control	0	2.1×10^5^ a^d^	3.1×10^5^ a
Fosthiazate	0.1	1.7×10^5^ b	2.1×10^5^ b
	0.5	3.2×10^4^ c	4.1×10^4^ c
	1.0	1.2×10^4^ cb	8.2×10^3^ cd
	3.0	3.2×10^3^ d	2.1×10^2^ d
	5.0	- ^e^	-
Ethoprophos	0.15	1.7×10^5^ a	2.1×10^5^ b
	0.5	2.4×10^4^ b	4.2×10^4^ c
	1.0	5.2×10^3^ c	8.9×10^3^ d
	3.0	8.6×10^2^ c	4.1×10^2^ d
	5.0	-	-
Terbufos	0.1	1.6×10^5^ a	7.8×10^4^ b
	0.5	5.1×10^4^ b	2.6×10^4^ c
	1.0	2.4×10^4^ c	4.1×10^3^ d
	3.0	3.2×10^3^ c	2.2×10^2^ d
	5.0	-	-

^a^The strains were inoculated with 1.5×10^2^ in Bennett’s medium containing anti-nematode agents. After the bacteria were incubated at 28°C for 72 h, their growth was measured. The experiment was performed with five replicates.

^b^Fosthiazate: Seonchungtan; Ethoprophos: Mocap; Terbufos: Kaunta; Control: no treatment.

^c^This column refers to the concentrations of anti-nematode agents in the Bennett’s medium.

^d^Values with the same letter in a column do not significantly differ (*p* < 0.05) based on Duncan’s multiple range test.

^e^Microorganisms were not detected.

**Table 2:** Mean growth characteristics of red pepper plants treated with *S. misionensis* KRA-24 and *S. yatensis* KRA-28 in pot experiments.^a^

Treatment ^b^	30 days				60 days			
	
	Root length (cm)	Shoot length (cm)	Dry root weight (g)	Dry shoot weight (g)	Root length (cm)	Shoot length (cm)	Dry root weight (g)	Dry shoot weight (g)
Control	18.8 ± 0.8 a ^c^	24.4 ± 1.5 c	0.17 ± 0.01 abc	0.57 ± 0.03 c	20.1 ± 1.5 b	28.4 ± 1.1 b	0.23 ± 0.01 a	0.65 ± 0.04 d
KRA-24	18.6 ± 0.9 a	28.6 ± 1.1 ab	0.17 ± 0.01 cb	0.66 ± 0.05 b	20.1 ± 1.1 b	30.0 ± 2.3 ab	0.19 ± 0.01 b	0.62 ± 0.01 d
KRA-28	19.0 ± 0.7 a	29.2 ± 1.3 a	0.17 ± 0.01 cb	0.78 ± 0.03 a	22.0 ± 1.6 b	32.8 ± 2.4 a	0.22 ± 0.03 ab	0.83 ± 0.06 b
F	18.6 ± 1.1 a	27.2 ± 1.6 ab	0.16 ± 0.02 c	0.52 ± 0.09 c	20.0 ± 1.5 b	32.2 ± 0.8 a	0.15 ± 0.02 c	0.47 ± 0.05 e
F + KRA-24	10.0 ± 2.9 a	28.6 ± 1.8 ab	0.19 ± 0.01 a	0.65 ± 0.05 b	21.1 ± 2.6 b	30.1 ± 2.5 ab	0.20 ± 0.03 ab	0.74 ± 0.05 c
F + KRA-28	18.4 ± 2.1 a	28.0 ± 1.5 ab	0.18 ± 0.02 ab	0.69 ± 0.05 b	22.4 ± 2.7 b	31.4 ± 4.5 ab	0.21 ± 0.03 ab	0.76 ± 0.07 c
H_2_O ^d^	19.6 ± 1.1 a	26.6 ± 1.7 b	0.17 ± 0.01 cb	0.83 ± 0.02 a	31.8 ± 2.4 a	32.8 ± 2.2 a	0.21 ± 0.01 ab	0.92 ± 0.03 a

^a^Each pot was watered using 100 ml/pot every 4 days, and culture broths were applied to plants at 100 ml/pot every 2 weeks. After 30 and 60 days, the length and dry weight of roots and shoots were measured.

^b^Control: treatment with GSS medium without microbial culture; KRA-24 and KRA-28: treatment with culture broth of *S. misionensis* KRA-24 and *S. yatensis* KRA-28; F: treatment of fosthiazate; F+KRA-24 and F+KRA-28: combination of fosthiazate and KRA-24 or KRA-28.

^c^Values with the same letter in a column do not significantly differ (*p* < 0.05) based on Duncan’s multiple range test.

^d^The plants were not infected with J2s and were only treated with water.

**Table 3:** Occurrence and propagation of *Meloidogyne incognita* in red pepper root and soil in pot experiments.

Treatment ^a^	1 day	30 days		60 days	
		
	No. of inoculated juveniles	Juveniles per 100 g soil	Egg masses per root ^b^	Juveniles per 100 g soil	Egg masses per root
Control	1032 ± 74.6 a ^c^	1286 ± 56.4 a	84.8 ± 5.8 a	1836 ± 84.4 a	142.8 ± 5.3 a
KRA-24	1006 ± 34.4 a	520 ± 91.4 b	62.6 ± 8.5 b	644 ± 32.9 b	73.8 ± 4.6 b
KRA-28	1018 ± 63.4 a	360 ± 57.9 c	50.02 ± 4.8 c	542 ± 48.2 bc	42.2 ± 4.8 c
F	996 ± 24.1 a	294 ± 113.9 cd	6.8 ± 2.6 d	492 ± 135.9 c	14.4 ± 3.9 d
F + KRA-24	996 ± 20.7 a	272 ± 83.5 cd	6.2 ± 3.6 d	504 ± 118.0 c	15.8 ± 3.1 d
F + KRA-28	1004 ± 81.4 a	240 ± 50.0 d	6.4 ± 2.1 d	490 ± 80.6 c	10.08 ± 3.4 d
H_2_O ^d^	-	-	-	-	-

^a^Control: treatment with GSS medium without microbial culture; KRA-24 and KRA-28: treatment with culture broth of *S. misionensis* KRA-24 and *S. yatensis* KRA-28; F: treatment of fosthiazate; F+KRA-25 and F+KRA-28: combination of fosthiazate and KRA-24 or KRA-28.

^b^The number of egg masses on roots was determined using the Phloxine B staining method.

^c^Values with the same letter in a column do not significantly differ (*p* < 0.05) based on Duncan’s multiple range test.

^d^The plants were not infected with J2s and were only treated with water.

^e^Juveniles were not detected.

**Table 4:** The change in microorganism abundances in soil.^a^

Treatment ^b^	1 day before treatment c		30 days		60 days	
		
	Total bacteria per 1 g soil	Actinomycetes per 1 g soil	Total bacteria per 1 g soil	Actinomycetes per 1 g soil	Total bacteria per 1 g soil	Actinomycetes per 1 g soil
Control	4.9 ± 0.9 ×10^3^ a ^d^	- ^e^	6.2 ± 1.3 ×10^4^ a	-	6.7 ± 2.5 ×10^5^ a	-
KRA-24	4.0 ± 2.2 ×10^3^ a	-	2.6 ± 1.3 ×10^3^ c	5.0 ± 1.7 ×10^3^ a	3.6 ± 2.0 ×10^4^ b	5.1 ± 0.9 ×10^4^ b
KRA-28	5.4 ± 1.1 ×10^3^ a	-	2.9 ± 1.2 ×10^3^ c	7.0 ± 1.9 ×10^3^ a	6.5 ± 2.9 ×10^4^ b	7.1 ± 0.9 ×10^4^ ab
F	4.6 ± 1.8 ×10^3^ a	-	3.9 ± 1.9 ×10^2^ c	-	5.8 ± 3.4 ×10^3^ b	-
F + KRA-24	5.2 ± 2.0 ×10^3^ a	-	5.4 ± 3.9 ×10^2^ c	5.9 ± 1.6 ×10^3^ a	2.4 ± 1.2 ×10^3^ b	7.0 ± 2.5 ×10^4^ ab
F + KRA-28	4.3 ± 1.5 ×10^3^ a	-	6.2 ± 2.6 ×10^2^ c	7.2 ± 0.7 ×10^3^ a	2.9 ± 1.3 ×10^3^ b	8.3 ± 1.2 ×10^4^ a
H_2_O	4.3 ± 2.1 ×10^3^ a	-	2.7 ± 1.5 ×10^4^ b	-	6.7 ± 1.5 ×10^4^ b	-

^a^The number of bacteria in the soil, including actinomycetes, was determined using the modified funnel method.

^b^Control: treatment with GSS medium without microbial culture; KRA-24 and KRA-28: treatment with culture broth of *S. misionensis* KRA-24 and *S. yatensis* KRA-28; F: treatment of fosthiazate; F+KRA-25 and F+KRA-28: combination of fosthiazate and KRA-24 or KRA-28.

^c^The number of microorganisms before treatment with *S. misionensis* KRA-24 and *S. yatensis* KRA-28 culture broth.

^d^Values with the same letter in a column do not significantly differ (*p* < 0.05) based on Duncan’s multiple range test.

^e^Actinomycetes were not detected.
